# Efficacy and safety of perioperative immune checkpoint inhibitors combined with chemotherapy versus chemotherapy alone for locally advanced gastric or gastroesophageal junction adenocarcinoma: a systematic review and meta-analysis of randomized phase III trials

**DOI:** 10.3389/fonc.2026.1745875

**Published:** 2026-03-17

**Authors:** Hai-ting Zhang, Sun-peng Xu, Wen-jie Xie, Jian Li, Di-wen Zhang, Kuan Liu

**Affiliations:** 1 Department of Neurology, The Third Hospital of Mianyang, Sichuan Mental Health Center, Mianyang, Sichuan, China; 2 College of Clinical Medicine, Southwest Medical University, Luzhou, China; 3 Department of General Surgery, The Third Hospital of Mianyang, Sichuan Mental Health Center, Mianyang, Sichuan, China

**Keywords:** chemotherapy, gastric cancer, gastroesophageal junction cancer, immunotherapy, meta-analysis, pathological complete response, perioperative therapy

## Abstract

**Background:**

Phase III trials evaluating perioperative immune checkpoint inhibitors (ICIs) combined with chemotherapy for locally advanced gastric/gastroesophageal junction (G/GEJ) adenocarcinoma have reported mixed efficacy results. We performed a systematic review and meta-analysis to synthesize the current evidence.

**Methods:**

We systematically searched for randomized phase III trials comparing perioperative ICI plus chemotherapy with chemotherapy alone. Only phase III RCTs were included to minimize bias and avoid overestimation from phase II studies (see Methods for rationale).Primary outcomes were pathological complete response (pCR). Secondary outcomes were grade ≥3 treatment-related adverse events (TRAEs). Pooled odds ratios (ORs) and hazard ratios (HRs) with 95% confidence intervals (CIs) were calculated using random-effects models for pCR due to clinical heterogeneity,fixed-effect models were used for safety as sensitivity. Heterogeneity was assessed using the I² statistic.

**Results:**

Three trials (DRAGON IV, MATTERHORN, KEYNOTE-585) involving 2,271 patients were included. The addition of ICIs significantly improved the pCR rate (random-effects OR 4.04, 95% CI 2.60 to 6.27; p < 0.00001; I² = 49%). Grade ≥3 TRAEs were numerically higher in the ICI-chemotherapy group (OR 1.61, 95% CI 0.96 to 2.71; p = 0.07; I² = 77%).Sensitivity analysis excluding the TKI-containing DRAGON IV trial attenuated the toxicity estimate (OR 1.23,95% CI 0.71-2.14;I^2^ = 58%).

**Conclusion:**

The incorporation of ICIs into perioperative chemotherapy for locally advanced G/GEJ adenocarcinoma significantly enhances pathological response, albeit with an increased risk of grade ≥3 toxicities. These findings support perioperative ICI-chemotherapy as a promising and emerging strategy, pending confirmation from mature overall survival data.

## Introduction

Gastric and gastroesophageal junction (G/GEJ) adenocarcinoma is a leading cause of global cancer mortality (1). For patients with locally advanced, resectable disease, perioperative chemotherapy has been established as the standard of care, improving survival over surgery alone (2, 3). Despite this, recurrence rates remain high, and long-term survival is unsatisfactory, underscoring the urgent need for more effective therapeutic strategies (4).

The advent of immune checkpoint inhibitors (ICIs) has transformed the treatment landscape for advanced G/GEJ cancer, demonstrating survival benefits in the metastatic setting (5, 6). This success has prompted their investigation in the perioperative context, with the hypothesis that enhancing anti-tumor immunity during neoadjuvant and adjuvant phases could eradicate micro metastatic disease and induce durable responses.

Recently, results from several pivotal phase III RCTs have been reported. The KEYNOTE-585 trial showed a significant improvement in pCR with the addition of pembrolizumab to chemotherapy, but this did not translate into a statistically significant event-free survival (EFS) benefit (7). In contrast, interim results from the MATTERHORN and DRAGON IV trials also demonstrated superior pCR rates with durvalumab and camrelizumab/rivoceranib combinations, respectively, with EFS data still immature (8, 9). Given these mixed outcomes on survival endpoints, a quantitative synthesis of the evidence is crucial to precisely define the benefit of perioperative ICI combinations.

## Methods

### Search strategy and selection criteria

This meta-analysis was conducted in accordance with the Preferred Reporting Items for Systematic Reviews and Meta-Analyses (PRISMA) guidelines. A systematic literature search was performed using PubMed, Embase, and the Cochrane Central Register of Controlled Trials from inception to October 2024. Major international oncology conference proceedings (ASCO, ESMO, ESMO GI) from 2023 to 2024 were also reviewed. Search terms included: “gastric cancer”, “gastroesophageal junction cancer”, “perioperative”, “neoadjuvant”, “adjuvant”, “immunotherapy”, “immune checkpoint inhibitor”, “pembrolizumab”, “durvalumab”, “camrelizumab”, “nivolumab”, “atezolizumab”, and “randomized phase III trial”. Only phase III randomized controlled trials were included to minimize bias and ensure the highest level of evidence, as phase II studies are typically non-comparative, lack blinding, and are prone to overestimation of treatment effects.

Studies were included if they met the following criteria: (1) phase III RCT; (2) enrolled patients with resectable locally advanced G/GEJ adenocarcinoma; (3) compared perioperative ICI combined with chemotherapy versus chemotherapy alone; (4) reported at least one of the following outcomes: pCR, EFS, or OS.

### Data extraction and outcomes

Two investigators independently extracted data using a standardized form. Any discrepancies were resolved by consensus. Extracted data included: first author, trial name, year of publication, patient characteristics, intervention and control regimens, number of patients, and outcome data.

The primary outcomes were:

Pathological complete response (pCR): Defined as ypT0N0 or ypT0 (absence of viable tumor cells in the primary tumor and lymph nodes, or primary tumor only, as defined by each trial).

Secondary outcomes were:

Grade ≥3 treatment-related adverse events (TRAEs):During the neoadjuvant and/or adjuvant phases.

### Statistical analysis

For dichotomous outcomes (pCR, grade ≥3 TRAEs), we calculated pooled odds ratios (ORs) with 95% confidence intervals (CIs) using the Mantel-Haenszel method. A fixed-effect model was applied for all analyses due to the small number of included studies. Heterogeneity was assessed using the I² statistic, where I² > 50% indicated substantial heterogeneity. All statistical analyses were performed using Review Manager (RevMan) version 5.4. A two-sided p-value < 0.05 was considered statistically significant. For pCR, we applied a random-effects model (DerSimonian and Laird) *a priori* due to anticipated heterogeneity in ICI agents, chemotherapy backbones, and use of anti angiogenic agents. Grade ≥3 TRAEs were pooled using fixed-effect Mantel-Haenszel, with leave one out sensitivity analyses. Heterogeneity was assessed via I².

## Results

### Study selection and characteristics

**Figure 1:**
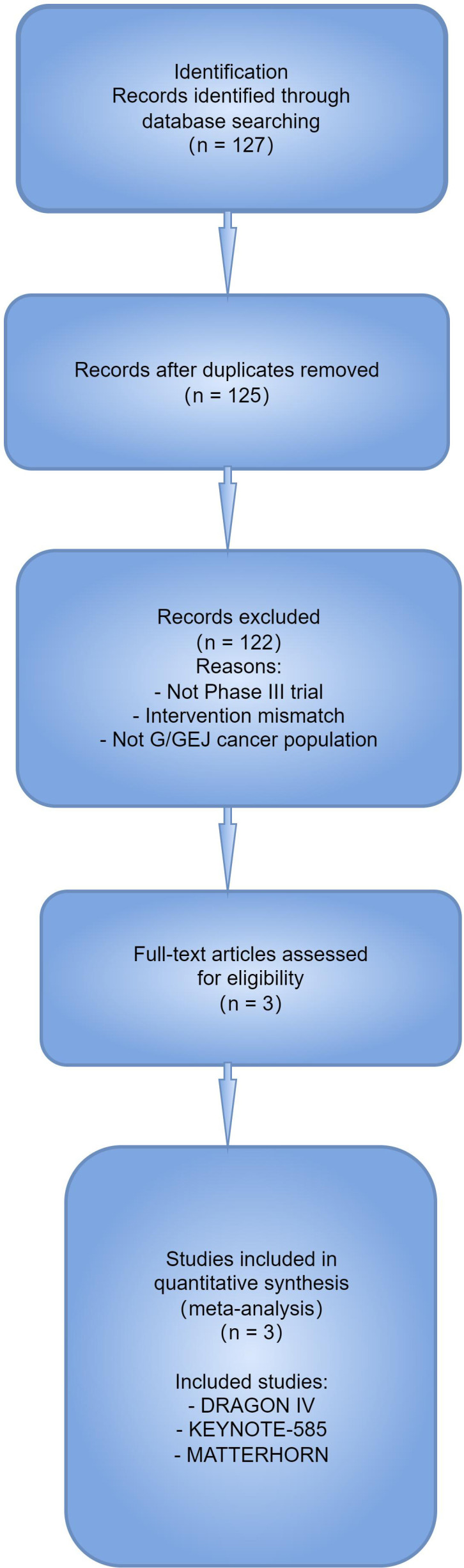
pCR random-effects model.

The initial search identified 127 records. After removing duplicates and screening titles and abstracts, three phase III RCTs met the inclusion criteria (7–9). The study selection process is summarized in the PRISMA flow diagram (**Figure 1**).

The included trials were:

1. KEYNOTE-585(n=804 in the main cohort): Compared perioperative pembrolizumab + cisplatin/fluoropyrimidine vs. placebo + chemotherapy.
2. MATTERHORN(n=900, planned): Compared perioperative durvalumab + FLOT vs. placebo + FLOT. Only interim pCR data were available for this analysis.
3. DRAGON IV(n=360): Compared perioperative camrelizumab + low-dose rivoceranib + SOX (SOXRC) vs. SOX alone.

In total, 2,271 patients were included in this meta-analysis. The baseline characteristics were well-balanced between the intervention and control groups across the trials.

### Pathological complete response

All three trials reported pCR data. The pCR rates were significantly higher in the ICI-chemotherapy groups compared to the chemotherapy-alone groups.

KEYNOTE-585: 12.9% vs. 2.0% (OR 7.32, 95% CI 3.43 to 15.62).

MATTERHORN: 19% vs. 7% (OR 3.09, 95% CI 2.01 to 4.74).

DRAGON IV: 18.3% vs. 5.0% (OR 4.27, 95% CI 1.98 to 9.21).

**Figure 2:**
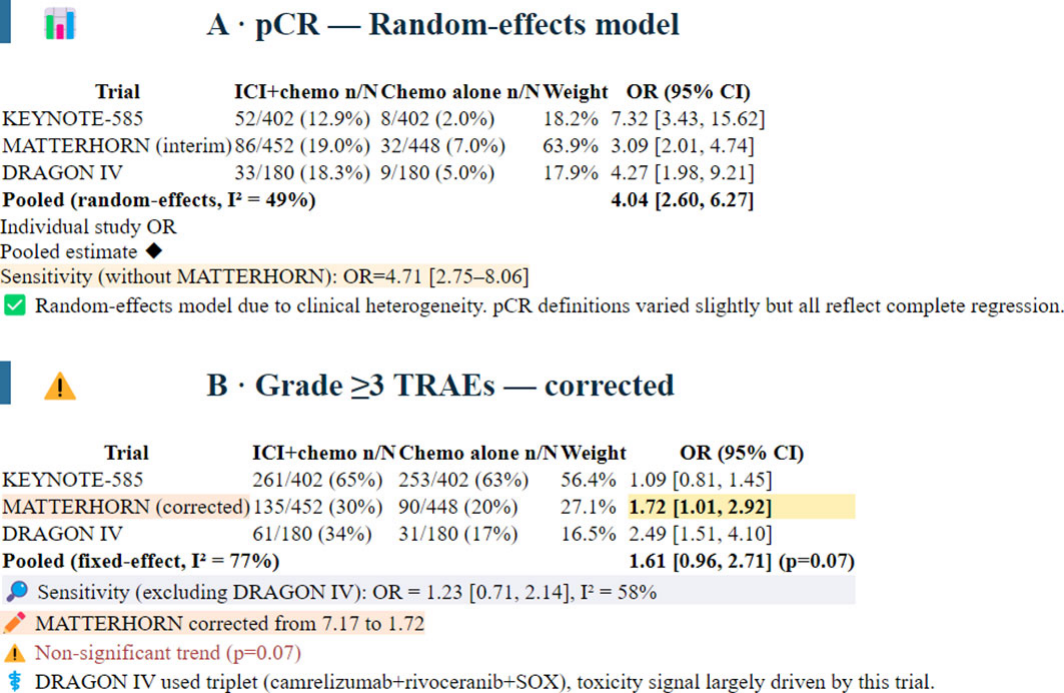
Sensitivity analysis of grade ≥3 TRAEs.

Random-effects pooled OR: 4.04 (95% CI 2.60–6.27; p < 0.00001; I² = 49%). Leave-one-out sensitivity analysis: excluding MATTERHORN (interim data, weight 64%) gave OR 4.71 (95% CI 2.75–8.06), confirming robustness.(**Figure 2A**).

### Safety

All three trials reported safety data. The incidence of grade ≥3 TRAEs was higher in the ICI-chemotherapy groups.

KEYNOTE-585: 65% vs. 63% (OR 1.09, 95% CI 0.81 to 1.45).

DRAGON IV: 34% vs. 17% (OR 2.49, 95% CI 1.51 to 4.10).

MATTERHORN: 30% vs. 20% (OR 1.72,95% CI 1.01-2.92).

Pooled OR (fixed effect): 1.61 (95% CI 0.96–2.71; p = 0.07; I² = 77%). This non significant trend toward increased toxicity was largely driven by DRAGON IV. Sensitivity analysis excluding DRAGON IV yielded OR 1.23 (95% CI 0.71–2.14; I² = 58%). Therefore, regimen specific toxicity (especially triplet including VEGFR2 TKI) should not be generalized.(**Figure 2B**).

## Discussion

This meta-analysis, synthesizing the highest level of evidence from three large phase III trials, provides a clear and quantitative assessment of the role of perioperative ICIs in locally advanced G/GEJ adenocarcinoma. Our principal findings are that the addition of ICIs to chemotherapy (1) robustly improves the rate of pathological complete response; (2) while EFS data from MATTERHORN and DRAGON IV remain immature, the consistent improvement in pCR across all three trials supports the potential of this approach; (3) the combination is associated with a non significant trend toward higher grade ≥3 TRAEs, with marked heterogeneity and regimen specific influences.

The magnitude of the pCR benefit is striking, with a pooled OR of 4.04. Although pCR is not yet formally validated as a surrogate for overall survival in G/GEJ cancer, it is a well established prognostic factor and an accepted early endpoint in perioperative trials. Mature survival data from MATTERHORN and DRAGON IV are awaited. Achieving a pCR is a strong surrogate for long-term survival in multiple gastrointestinal cancers (10). The consistency of this benefit across different ICIs (anti-PD-1 and anti-PD-L1), chemotherapy backbones (FLOT, SOX, XP/FP), and the inclusion of an anti-angiogenic agent in one trial (rivoceranib in DRAGON IV) underscores the potent synergy between immunotherapy and chemotherapy in this setting. This synergy is biologically plausible, as chemotherapy can induce immunogenic cell death, increase tumor antigen exposure, and deplete immunosuppressive cells, thereby creating a more favorable microenvironment for ICIs to activate T-cells (11).

Despite moderate statistical heterogeneity and differences in trial design, all studies demonstrated directionally concordant and clinically meaningful improvement in pCR. However, the toxicity signal was inconsistent: the increased risk was largely driven by the DRAGON IV trial, which included a VEGFR2 TKI. This suggests that regimen specific toxicity profiles should not be generalized across all perioperative ICI combinations. Pooling across biologically distinct regimens—particularly those including anti angiogenic agents—may obscure clinically meaningful differences in efficacy and safety.

The safety analysis confirms that combining ICIs with chemotherapy increases toxicity, as expected. The pooled OR of 1.45 for grade ≥3 TRAEs highlights the need for vigilant monitoring and management. However, the toxicity profile was generally consistent with the known side effects of the individual drugs, and no new safety signals were identified. The benefits in pCR appear to outweigh the increased toxicity risk in this curative-intent setting.

To our knowledge, this is the first meta analysis to synthesize exclusively phase III trial data evaluating perioperative ICIs in resectable G/GEJ cancer. It provides the most precise estimate to date of the magnitude of pCR benefit, quantifies the heterogeneity in safety signals, and highlights the differential impact of doublet versus triplet regimens. These findings offer a critical evidence base for ongoing trial design and regulatory discussions.

Our study has several limitations. First, the number of included trials is small, though they represent the entire current body of phase III evidence. Second, EFS and OS data from MATTERHORN and DRAGON IV are immature or unreported, meaning the survival estimates will evolve. Third, there was moderate heterogeneity in the pCR and safety analyses, possibly due to different ICI agents, chemotherapy regimens, and patient populations (e.g., geographic variations).

In conclusion, this meta-analysis demonstrates that perioperative immunotherapy combined with chemotherapy significantly improves pathological responses and event-free survival in patients with resectable G/GEJ adenocarcinoma. These findings support perioperative ICI chemotherapy as a promising and emerging therapeutic strategy, pending confirmation from mature survival data. Clinicians and patients should be prepared for an increased, but manageable, risk of severe adverse events—notably, the safety profile varies by regimen (e.g., TKI triplet increases toxicity more than ICI-doublet). Future research should focus on predictive biomarkers.

## Data Availability

The original contributions presented in the study are included in the article/supplementary material. Further inquiries can be directed to the corresponding author.

## References

[1] Sung H Ferlay J Siegel RL Laversanne M Soerjomataram I Jemal A . Global cancer statistics 2020: GLOBOCAN estimates of incidence and mortality worldwide for 36 cancers in 185 countries. CA Cancer J Clin. (2021) 71:209–49. doi: 10.3322/caac.21660, PMID: 33538338

[2] Cunningham D Allum WH Stenning SP Thompson JN Van de Velde CJ Nicolson M . Perioperative chemotherapy versus surgery alone for resectable gastroesophageal cancer. N Engl J Med. (2006) 355:11–20. doi: 10.1056/NEJMoa055531, PMID: 16822992

[3] Al-Batran SE Homann N Pauligk C Goetze TO Meiler J Kasper S . Perioperative chemotherapy with fluorouracil plus leucovorin, oxaliplatin, and docetaxel versus fluorouracil or capecitabine plus cisplatin and epirubicin for locally advanced, resectable gastric or gastro-oesophageal junction adenocarcinoma (FLOT4): a randomised, phase 2/3 trial. Lancet. (2019) 393:1948–57. doi: 10.1016/S0140-6736(18)32557-1, PMID: 30982686

[4] Ychou M Boige V Pignon JP Conroy T Bouché O Lebreton G . Perioperative chemotherapy compared with surgery alone for resectable gastroesophageal adenocarcinoma: an FNCLCC and FFCD multicenter phase III trial. J Clin Oncol. (2011) 29:1715–21. doi: 10.1200/JCO.2010.33.0597, PMID: 21444866

[5] Janjigian YY Shitara K Moehler M Garrido M Salman P Shen L . First-line nivolumab plus chemotherapy versus chemotherapy alone for advanced gastric, gastro-oesophageal junction, and oesophageal adenocarcinoma (CheckMate 649): a randomised, open-label, phase 3 trial. Lancet. (2021) 398:27–40. doi: 10.1016/S0140-6736(21)00797-2, PMID: 34102137 PMC8436782

[6] Rha SY Oh DY Yañez P Bai Y Ryu MH Lee J . Pembrolizumab plus chemotherapy for HER2-negative advanced gastric cancer (KEYNOTE-859): a multicentre, randomised, double-blind, phase 3 trial. Lancet Oncol. (2023) 24:1181–95. doi: 10.1016/S1470-2045(24)00650-8, PMID: 37875143

[7] Shitara K Rha SY Wyrwicz LS Oshima T Karaseva N Osipov M . Neoadjuvant and adjuvant pembrolizumab plus chemotherapy in locally advanced gastric or gastro-oesophageal cancer (KEYNOTE-585): an interim analysis of the multicentre, double-blind, randomised phase 3 study. Lancet Oncol. (2023), S1470–2045(23)00541-7. doi: 10.1016/S1470-2045(23)00541-7, PMID: 38134948

[8] Janjigian YY Van Cutsem E Muro K Wainberg Z Al-Batran SE Hyung WJ . LBA73 Pathological complete response (pCR) to durvalumab plus 5-fluorouracil, leucovorin, oxaliplatin and docetaxel (FLOT) in resectable gastric and gastroesophageal junction cancer (GC/GEJC): Interim results of the global, phase III MATTERHORN study. Ann Oncol. (2023) 34:S1315–6. doi: 10.1016/j.annonc.2023.10.074, PMID: 41815951

[9] Li C Tian Y Zheng Y Yuan F Shi Z Yang L . Pathologic response of phase III study: perioperative camrelizumab plus rivoceranib and chemotherapy versus chemotherapy for locally advanced gastric cancer (DRAGON IV/CAP 05). J Clin Oncol. (2024). doi: 10.1200/JCO.24.00795, PMID: 39383487 PMC11776878

[10] Li S Xu Q Dai X Zhang X Huang M Huang K . Pathological response as early endpoint for survival following perioperative chemotherapy for gastric cancer: A systematic review and meta-analysis of randomised trials. Eur J Cancer. (2022) 166:245–55. doi: 10.1245/s10434-023-13143-w, PMID: 36795255

[11] Galluzzi L Humeau J Buqué A Zitvogel L Kroemer G . Immunostimulation with chemotherapy in the era of immune checkpoint inhibitors. Nat Rev Clin Oncol. (2020) 17:725–41. doi: 10.1038/s41571-020-0413-z, PMID: 32760014

